# Antibacterial Films Made of Bacterial Cellulose

**DOI:** 10.3390/polym14163306

**Published:** 2022-08-13

**Authors:** Zhenbing Sun, Xiaoping Li, Zhengjie Tang, Xiaobao Li, Jeffrey J. Morrell, Johnny Beaugrand, Yao Yao, Qingzhuang Zheng

**Affiliations:** 1 Yunnan Key Laboratory of Wood Adhesives and Glue Products, Southwest Forestry University, Kunming 650224, China; 2 International Joint Research Center for Biomass Materials, Southwest Forestry University, Kunming 650224, China; 3 National Centre for Timber Durability and Design Life, University of the Sunshine Coast, Brisbane, QLD 4102, Australia; 4 Biopolymères Interactions Assemblages (BIA), INRA, Rue de la Géraudière, F-44316 Nantes, France

**Keywords:** carboxymethyl bacterial cellulose (CMBC), nano-Ag, nano-bacterial cellulose (NBC), mechanical properties, pyrolysis characteristics

## Abstract

Bacterial cellulose (BC) is naturally degradable, highly biocompatible, hydrophilic, and essentially non-toxic, making it potentially useful as a base for creating more sophisticated bio-based materials. BC is similar to plant-derived cellulose in terms of chemical composition and structure but has a number of important differences in microstructure that could provide some unique opportunities for use as a scaffold for other functions. In this study, bacterial cellulose was alkylated and then esterified to produce a carboxymethyl bacterial cellulose (CMBC) that was then used to produce six different composite films with potential antibacterial properties. The films were assessed for antibacterial activity against *Staphylococcus aureus* and *Escherichia coli*, pyrolysis characteristics using thermogravimetric analysis (TGA), microstructure using scanning electron microscopy (SEM), and mechanical properties. The addition of nano-silver (nano-Ag) markedly improved the antimicrobial activity of the films while also enhancing the physical and mechanical properties. The results indicate that the three-dimensional reticulated structure of the bacterial cellulose provides an excellent substrate for scaffolding other bioactive materials. Thus, the nano-BC was added into the CMBC/nano-Ag composites furthermore, and then the antibacterial and mechanical properties were improved 44% for *E. coli*, 59% for *S. aureus*, and 20% for tensile strength, respectively.

## 1. Introduction

Bacterial cellulose (BC) is known for its desirable properties, including sustainability, biocompatibility, biodegradability, extensive chemical modification capabilities, and high surface area [1,2,3].

Many BC composites also have antimicrobial properties, but can be naturally degraded, making them a potentially recyclable packaging raw material. However, BC is not anti-bacterial and must be modified to create microbially resistant materials [4]. For example, curcumin (Cur) microspheres and nano-particles were formed in situ in a BCNF/CNF network to create an antimicrobial film. The nanofibers were associated with more uniform curcumin distribution; however, antimicrobial activity was less pronounced and needed further improvement. Other researchers have incorporated chitosan (CS) and chitosan oligomer (COS) into a bacterial cellulose matrix produced films that killed over 99.9% of *E. coli* and *S. aureus* cells, while incorporating chitosan oligomers resulted in 95.6 and 99.6% of *E. coli* and *S. aureus* killed cells, respectively. The BC-COS composite films also exhibited good tensile strength and elongation at break of 9.67 ± 0.26 MPa and 1.87 ± 0.15%, respectively, indicating that BC-COS composite films would be more suitable for food packaging films, although there might be a need for increased tensile strength depending on the application [5]. Nano-bacterial cellulose could be used as a good performance dispersant and adsorbent because of its nanostructure. Therefore, in this study, bacterial nano-cellulose was added to the composite film to make the antimicrobial agent uniformly dispersed in the composite film.

Many studies have been conducted on carboxymethyl cellulose (CMC) antibacterial composite films [6,7,8,9,10]. A broad-spectrum antibacterial CMC composite film was prepared by adding different proportions of okra mucilage and zinc oxide nano-particles.

The optimal formulation inhibited the growth of both *S. aureus* and *E. coli* with a tensile strength of 24.22 ± 0.58 MPa [11]. CMC/chitosan composites have been used as an edible fruit packaging and exhibit excellent activity against *Salmonella* and *E. coli* [12]. These studies suggest the need for further research to identify antimicrobial composite films that retain their mechanical properties. Bacterially-derived cellulose may provide a more suitable substrate for this effort.

This research explored the effects of coupling bacterial cellulose with two essential plant oils and four different nano-particles (rose essential oil, cumin essential oil, nano-silica, nano-titanium dioxide, nano-silver, and nano-bacterial cellulose) on both the antimicrobial and mechanical properties of the resulting films.

## 2. Materials and Methods

### 2.1. Bacterial Cellulose (BC) and Cellulose from Plant Preparation

**Figure 1:**
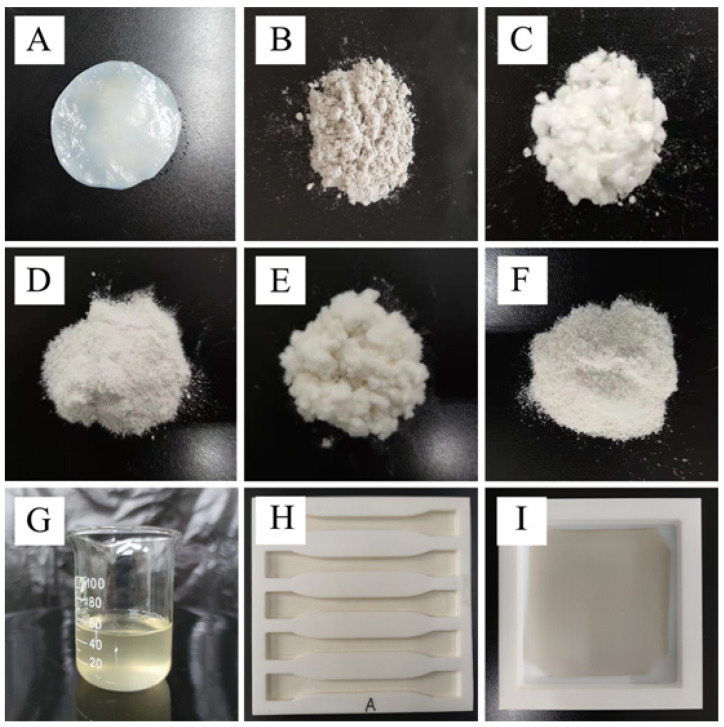
Examples of (**A**) Wet BC, (**B**) Dry BC powder, (**C**) Cellulose-C, (**D**) Cellulose-H (**E**) Cellulose-B, (**F**) CMBC, (**G**) CMBC solution, (**H**,**I**) CMBC composite films.

BC from *Taonella mepensis* was supplied by Beinacruz Biotechnology Co. Ltd., (Suzhou, Jiangsu, China). The bacteria were grown for 7 days on a medium containing 20.0 g glucose, 5.0 g yeast paste, 1.0 g K_2_HPO_4_, 15.0 g MgSO_4_, and 5 mL anhydrous ethanol per L distilled water (pH 4.5). The resulting bacterial mat was soaked in distilled water which was changed hourly for 12 h to remove residual media. The wet mat was cut into 3 cm discs that were soaked for one hour in 1% sodium hydroxide at 80 °C before being rinsed in distilled water that was changed every 2 h until the solution pH was 7.0 (Figure 1A). The resulting bacterial cellulose gel was drained and vacuum dried for 72 h at 80 °C before being crushed into a powder (60 mesh screen) using a high-speed grinder (Figure 1B).

Plant-based cellulose was obtained from *Cryptomeria* *ortune* (Cellulose-C), hemp (Canabis sativa) hurd (Cellulose-H), and bamboo (*Dendrocalamus giganteus*) (Cellulose-B) obtained from locally grown materials (Kunming, Yunnan, China). The material was ground to pass a 40-mesh mesh screen and soaked in an excess of 95% ethanol for 6 h at 70 °C to remove fatty acids. The samples were treated with glacial acetic acid and sodium chlorite at 75 °C for 4 h to remove the lignin and then treated with 17.5% NaOH at room temperature for 45 min to remove the hemicelluloses. The samples were neutralized by repeated washing with distilled water before being dried at 104 °C (Shown in Figure 1C–E, respectively).

### 2.2. Carboxymethyl Cellulose Bacterial Cellulose (CMBC) and CMC Preparation

8g BC or cellulose derived from plants (*Cryptomeria fortunei*, hemp hurd and bamboo), 160 mL of 95% ethanol, and 40 mL of 30% NaOH solution were mixed and stirred for 60 min at 30 °C. Then, 10 g of sodium chloroacetate were added and the temperature was increased to 65 °C and stirred for 3 h. Glacial acetic acid (90%) solution was added to reduce the pH of the mixture and then the samples were washed with alcohol until the pH was 7. The neutralized samples were oven-dried at 65 °C and stored for later use (Figure 1F).

### 2.3. Characterizations of BC and CMBC

Degree of Substitution (*DS*): The degree of substitution of the hydroxyl group has an important influence on the resulting CMC properties. The degree of substitution was determined by the acidimeter method by weighing 0.2 g (accuracy 0.1 mg) of the sample, dissolving it in 80 mL of water, stirring electromagnetically for 10 min, and adjusting the pH of the solution to 8.0. The sample was titrated with continuous stirring using a standard titration solution of sulphuric acid until the pH was 3.74. The number of mL of sulphuric acid titration solution used was recorded (to the nearest 0.05 mL). The degree of substitution (*DS*) was then calculated using the amount required to reach the end point according to Equations (1) and (2), as follows. Five copies were performed for each sample.

(1) $$B=\frac{2cV}{m}$$

(2) $$DS=\frac{0.162B}{1-0.08B}$$

where *B* = amount of carboxymethyl substance contained in the sample, mmol/g;

*m* = Quality of the sample, g;

*c* = Concentration of sulfuric acid standard titration solution, mol/L;

*V* = Volume value of standard titration solution of sulfuric acid, mL;

Microstructure: The samples were placed on an aluminum grid and examined by field emission scanning electron microscopy on a Nova Nano SEM450 microscope (FEI, Hillsboro, OR, USA). At least five fields were examined for each sample.

Fourier Transform Infrared Spectroscopy (FTIR): The samples were mixed with KBr, pressed into a pellet, and analyzed on a Nicolet i50 FTIR Analyzer (Thermo Scientific, Waltham, MA, USA). Samples were subjected to 64 scans and the resulting spectra were baseline corrected and then analyzed for differences in spectra for different raw materials.

X-ray diffraction (XRD) analysis: The samples were examined by X-ray diffractometry on a Rigaku Ultima IV X-ray diffractometer (Rigaku Corp, Tokyo, Japan) (XRD, Ulti) using a scanning angle from 10° to 40°, a step size of 0.026° (accelerating current = 30 mA and voltage = 40 kV), and Cu-Kα radiation of λ = 0.154 nm.

Thermogravimetric (TG) analysis: Approximately 5.0 to 6.0 mg of the samples were ground to pass an 80-mesh to 120-mesh and placed into sample holders for analysis on a TGA92 thermo gravimetric analyzer (KEP Technologies EMEA, Caluire, France). N_2_ was used as the shielding gas and Al_2_O_3_ as the reference compound. The temperature was increased from room temperature (approx. 20–23 °C) to 600 °C at a rate of 20 °C/min to produce thermogravimetric curves.

### 2.4. Preparation of CMBC and CMC Composite Films

A 1.5% solution of a given film was prepared by adding 98.5 mL distilled water, 1.5 g CMBC or CMC in a heated magnetic mixer at 900–1000 rpm at 45 °C. When the CMC was completely dissolved in water, 0.8 g of sodium alginate and 0.25 g of glycerin were added and stirred until the mixture was uniform, then the temperature was raised to 70 °C until the sodium alginate and glycerin were entirely dissolved, a further 4.5 mg of Rose essential oil, Cumin essential oil, Nano-silver (Nano-Ag, particle diameter of 60–80 nm), nano-silica dioxide (Nano-SiO_2_, particle diameter of 30 ± nm), nano-titanium dioxide (Nano-TiO_2_, particle diameter of 25 ± nm), or 0.58 g nano-bacterial cellulose (nano-BC, particle diameter of 40 ± nm) solution (prepared by biological enzymes and mechanical mixing in Lab of Yunnan Key Laboratory of Wood Adhesives and Glue Products, with a solid content of 0.26% [13]) were added, then the film solution was placed in an ultrasonic cleaner at 50 Hz for 12 min to remove the air bubbles. The solution (Figure 1G) was then cast on a PTFE mold and dried at 30 °C for 48 h (Figure 1H,I).

### 2.5. Antimicrobial Testing of CMBC Composite Films

The growth media contained 10.0 g of peptone, 3.0 g of beef paste, 5.0 g of sodium chloride, and 20 g of nutrient agar powder/liter of water. The disc diffusion method was used to assess the antibiotic activity of CMBC composite films against *S. aureus* and *E. coli* [14]. Disks (6 mm in diameter) cut from the sterilized composite films were placed on the medium and incubated at 37 °C and 70% relative humidity for 24 h. Antibacterial performance of the composite film was assessed by measuring the diameter of the inhibition circle around the sample to the nearest mm. Each material was assessed on disks per bacterium.

### 2.6. Tensile Properties, Opacity, Viscosity, and Water Vapor Permeability of Carboxymethyl Bacterial Cellulose Antibacterial Composite Film

Tensile strength (MPa) and elongation at break (%) were measured on 10 replicates of 0.089- to 0.098-mm by 150-mm-long dog-bone samples of each material on a Universal Testing Machine according to procedures described in GB/T 1040.1-2006 (Plastics Determination of tensile properties). A load was applied to failure at a rate of 1 mm/min. Five replicates were performed for each sample.

The opacity of the CMC composite films was tested by cutting 10- by 40-mm-long samples and placing them on the inner surface on one side of a cuvette and measuring absorbance at 600 nm on an XP Spectrum 752 ultraviolet spectrophotometer (XP-Spectrum Company, Shanghai, China). Five measurements were made for each sample [15].

The viscosity of the composite film solution was measured using a MARS60 Nicolay Rotational rheometer (MARS60, HAAKE Company, Vreden, Germany). Five replicates were performed for each sample.

Water vapor permeability indicates the ability of water vapor to pass through a material.

The water vapor transmission coefficient of the specimen was calculated according to Equation (1) [16].

(3) $$p=\frac{\Delta m\times d}{A\times t\times \Delta p}$$

where p is the water vapor transmission coefficient of the sample in grams/square centimeter per second Pascal g.cm/(cm^2^. s. Pa). Δ*m* is the change in the mass of the sample in grams (g) during the period *t*. *A* is the sample area through the water vapor in square meters (m^2^). *t* is the difference in time between two intervals after the mass change has stabilized in hours (h). *d* is the thickness of the specimen in centimeters (cm).

Δ*p* is the difference in water vapor pressure between the two sides of the specimen in Pascals (Pa). Five replicates were performed for each sample type.

## 3. Results and Discussion

### 3.1. Characteristics or Multi-Feature Exploration of BC and CMBC

#### 3.1.1. DS of CMBC and CMC

The degree of substitution (*DS*) of CMC is the average number of H substituted by (-CH_2_COONa) on the hydroxyl group (-OH) of each glucose monomer in the molecular structure of CMC. The theoretical maximum value can reach 3, and many studies have successfully prepared CMC with *DS* > 1 [17]. As *DS* increases, the transparency and solubility of the solution improve significantly. The *DS* of CMC prepared from *C. fortunei*, hemp hurd or bamboo cellulose were 0.64, 0.64, and 0.65, respectively, while the *DS* of CMBC was 0.81 (Table 1). CMC can be dissolved in an alkaline aqueous solution when the *DS* is >0.3, while CMC can be better integrated with plasticizers and thickeners when the *DS* is >0.7. This is a prerequisite for the preparation of high-performance CMC composite films, although it is important to note that *DS* of CMC can change depending on the test method [18].

#### 3.1.2. Micro-Structure of BC, Cellulose, CMBC, and CMC

**Figure 2:**
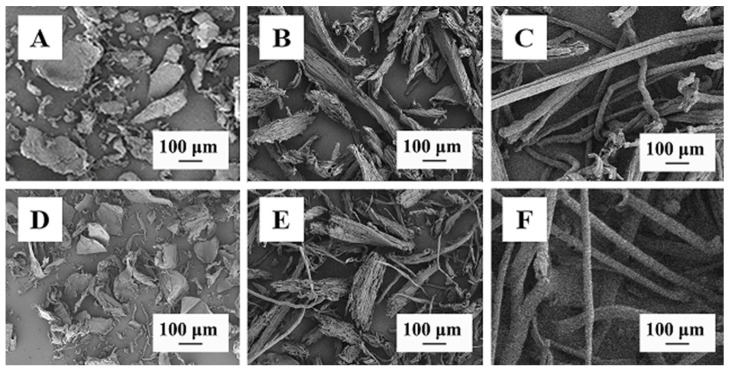
The microstructure BC (**A**), Cellulose-H (**B**), Cellulose-B (**C**), CMBC (**D**), CMC-C (**E**), and CMC-B (**F**).

The microstructure of BC, cellulose, CMBC, and CMC are shown in Figure 2. Cellulose source has a significant effect on cellulose shape; BC is lamellar (Figure 2A), the Hemp cellulose is a thick rod (Figure 2B), while bamboo cellulose is a slender rod (Figure 2C). Cellulose microstructure did not change substantially after it was transformed into CMC (Figure 2D–F), but surface roughness increased. This change may reflect the high concentration of (30%) sodium hydroxide used in the alkalization and etherification processes [19].

#### 3.1.3. FTIR Spectrum of BC, Cellulose, CMBC, and CMC

**Figure 3:**
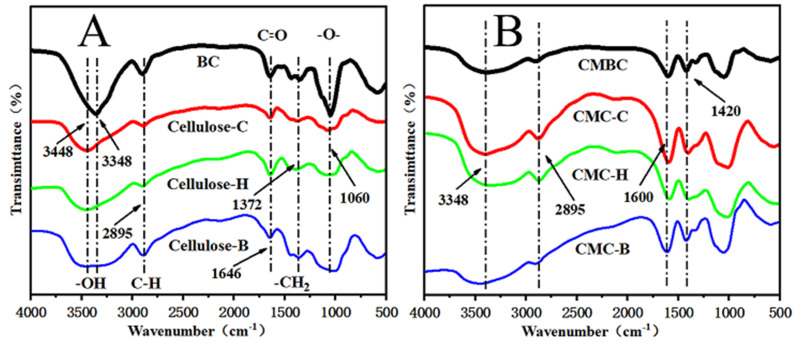
FTIR spectrum of (**A**) BC, cellulose-C, cellulose-H, cellulose-B, (**B**) CMBC, CMC-C, CMC-H, and CMC-B.

FTIR spectra of BC and cellulose from the three plant sources are shown in Figure 3A. The characteristic cellulose peaks at 3348 cm^−1^ (O–H Stretching vibration), 2895 cm^−1^ (C–H Stretching vibration), 1646 cm^−1^ (Conjugate C=O Stretching vibration), 1372 cm^−1^ (C–H bending vibration), and 1060 cm^−1^ (C–O Stretching vibration), respectively, were present in all four cellulose sources [20]. Characteristic BC peaks were sharp with narrow absorption bands. The characteristic CMC peaks were observed at 1600 cm^−1^ and 1420 cm^−1^ (Figure 3B), which were enhanced, indicating that the cellulose molecule was modified after carboxymethylation, indicating that this treatment of BC was successful for CMBC.

#### 3.1.4. XRD Analysis of BC, CMBC, and CMC

**Figure 4:**
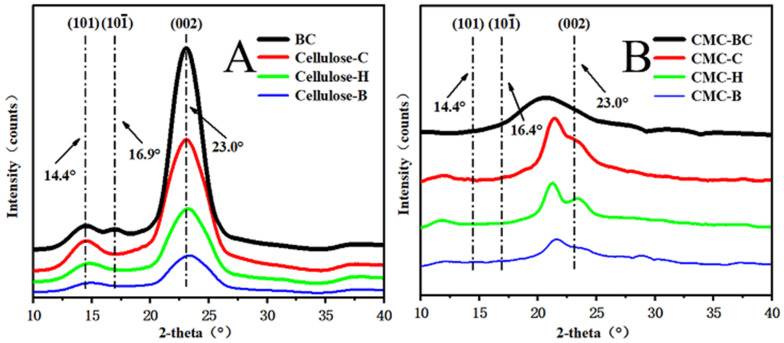
XRD spectrum of (**A**) BC and cellulose from *Cryptomeria fortunei* (Cellulose-C), Hemp hurd (Cellulose-H) and Bamboo (Cellulose-B), (**B**) CMBC, CMC of cellulose from *Cryptomeria fortunei* (CMC-C), Hemp hurd (CMC-H) and Bamboo (CMC-B).

The X-ray diffraction (XRD) analysis results for BC, cellulose, CMC, and CMBC are shown in Figure 4 at both (101) at 14.4°, and (002) at 23.0° [21]. BC and plant-derived celluloses had comparable crystalline Type 1 cellulose structures. However, CMBC and CMC from three plant sources only produced results at (002), indicating that sodium hydroxide transformed and reduced the crystal structure during etherification and alkylation. The CMBC and CMC were prepared successfully.

#### 3.1.5. The Pyrolysis Characteristics of BC and CMBC

**Figure 5:**
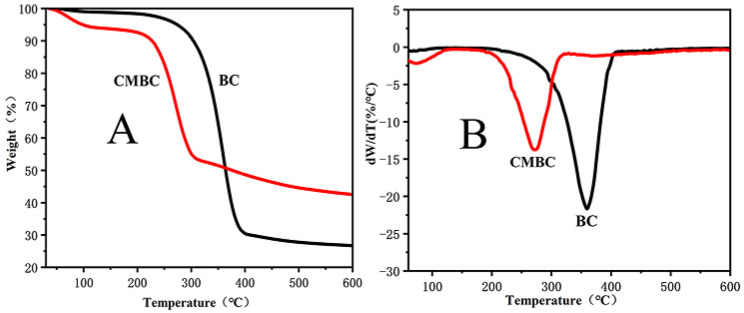
Thermogravimetric analysis (**A**) and differential thermogravimetric analysis (**B**).

The thermogravimetric (TG) curves for BC and CMBC are shown in Figure 5A. The residue rates of BC and BC-CMC were 26.6% and 43.3%, respectively, with two pyrolysis peaks (Figure 5B). The first peak occurred close to 100 °C and was related to moisture in raw materials. CMBC had a higher water content after carboxymethylation compared to BC. The second peak was more interesting as the main pyrolysis peak of BC occurred at 360 °C while the pyrolysis peak for CMBC was reduced to 270 °C. The CMBC appeared to be less thermostable, although it was still relatively resistant to thermal degradability, which could be an asset for packaging end uses.

### 3.2. Antibacterial, Microstructure, and Pyrolysis Characteristics Properties of Composite Films

**Figure 6:**
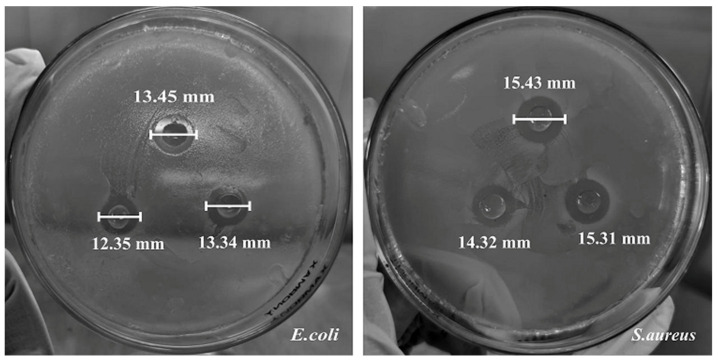
Effect of CMBC/Nano-Ag/NBC composite film against *S. aureus* and *E. coli*.

The diameter of bacterial inhibition was used as a measure of bacteriostatic ability of the modified cellulose composite films (Figure 6; Table 2). The clear zone around wells reflects the overall bacterial sensitivity [22]. The degree of inhibition around BC or plant cellulose against *E. coli* and *S. aureus* varied with the addition of two type essential oils and three types of nano-particles. Several researchers have found that rose essential oil contains high levels of phenolics, which exhibits vigorous antibacterial activity against *E. coli* and *S. aureus* [23]. However, nano-silver was less antibacterial, possibly because the film components inhibited movement. The control CMBC composite films and the CMBC composites with nano-silica had no inhibitory effect. The addition of nano-Ag particles into the CMBC composite films resulted in strong antibacterial activity, as evidenced by inhibition diameters of 9.04 and 9.41 mm for *E. coli* and *S. aureus*, respectively, possibly as the attachment of nano-Ag particles on the surface of the bacterial cell films, thereby interfering with cell permeability and respiratory function [24]. The nano-silver may also penetrate through the bacterial cell wall to disrupt normal metabolism [25,26]. The activities of nano-Ag particle amended CMC composite films created with the three plant cellulose sources tended to be lower than composite films made with BC. Thus, the BC based composite films were chosen for further exploration via addition of NBC. The degree of inhibition against *E. coli* and *S. aureus* increased significantly in the modified films (Figure 6), with increases of 44% and 59%, respectively. This indicates that NBC can better disperse and adsorb the nano-Ag in the composite film [27], thus improving the antibacterial effect of the composite film. In addition, it has been pointed out that cellulose fibers have a wide surface area, which means that the fibers can help the fibers adsorb nano-silver quite effectively through hydroxyl groups, thus making more nano-silver useful [28].

**Figure 7:**
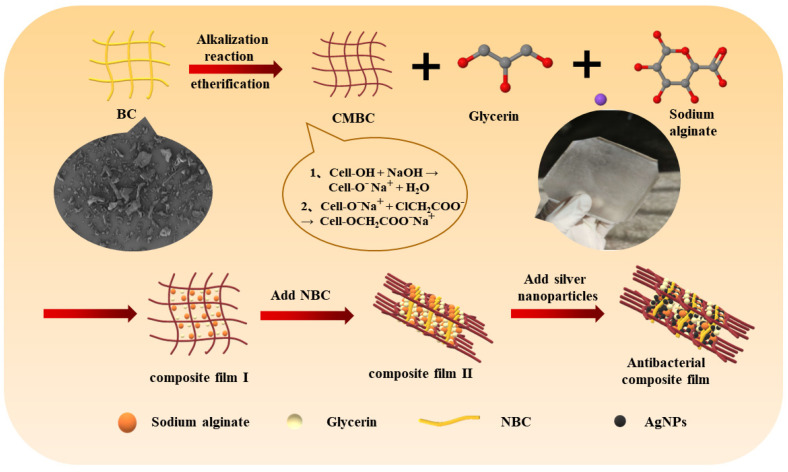
The preparation process of bacterial cellulose antibacterial composites and hypothesizes microstructural organization.

**Figure 8:**
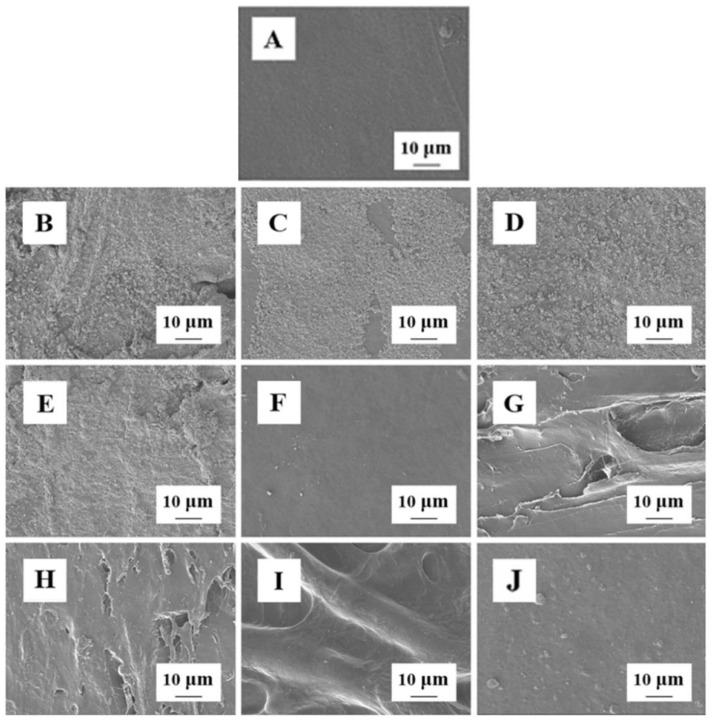
The microstructure composite films ((**A**) control; (**B**) Rose essential oil; (**C**) Fennel essential oil; (**D**) Nano-SiO_2_; (**E**) Nano-TiO_2_; (**F**) Nano-Ag; (**G**) CMC-C/nano-Ag; (**H**) CMC-H/nano-Ag; (**I**) CMC-B/nano-Ag; and (**J**) Nano-Ag/NBC, respectively).

The preparation process of the composite film is shown in Figure 7. The microstructure of composite films is shown in Figure 8. The addition of plant essential oils or nano-materials into the composite films was associated with surface roughening (Figure 8B–F). There were obvious cracks on the surfaces of CMC composite films composed of the three of plant-derived celluloses, possibly because of poor compatibility with the nano-Ag (Figure 8G–I). The film surface developed significant protrusions as NBC and nano-Ag were simultaneously added to the CMBC composite films, but the films remained intact (Figure 8J).

**Figure 9:**
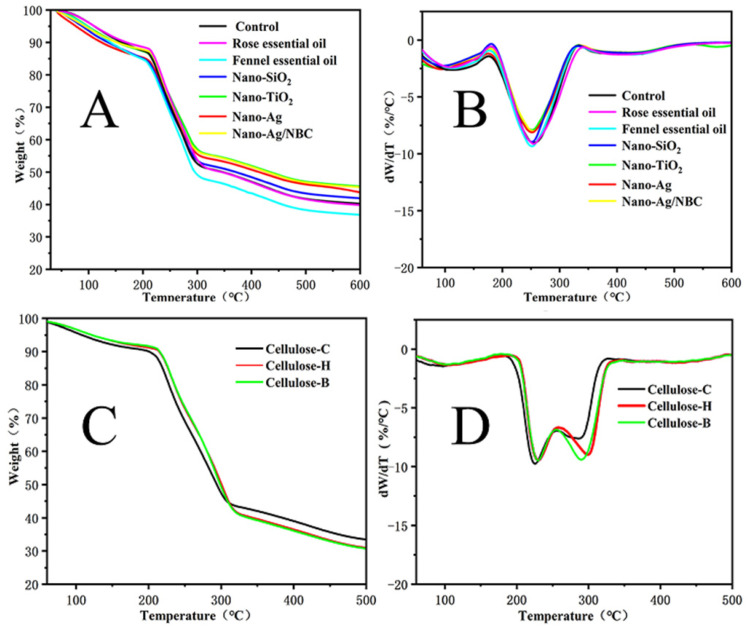
The pyrolysis curve of composite films ((**A**,**B**) for TG and DTG for CMBC composite films, respectively; (**C**,**D**) for TG and DTG for CMC composite with nano-Ag particles films, respectively).

The pyrolysis curves of composites (Figure 9), the residual material at 600 °C, and the peak temperature (Table 2) showed that pyrolysis peaks and pyrolysis residuals of composites films made from BC and plant cellulose differed significantly. The residual material from composites films made from plant-derived cellulose were 30.4% to 33.3%; lower than those for BC films (36.8% to 45.5%). Composite films made from BC had only one pyrolysis peak at about 250 °C, while composite films made from plant cellulose had two pyrolysis peaks, one at about 210 °C and the other at 300 °C. BC is homogeneous in terms of biochemical composition, whereas the plant-derived cellulose samples displayed a non-cellulosic degradation peak and a second higher peak corresponding to cellulose [29].

Composite films made from BC exhibited better properties than the plant-derived cellulose, including anti-bacterial activity, surface microstructure, and pyrolysis characteristic. In a word, the composite films made from BC have better characters compared with the plant cellulose, the characters including the inhibition, surface microstructure, and pyrolysis characters. Therefore, we will only discuss the mechanical properties of composites films from BC as followed.

### 3.3. Mechanical and Physical Properties of CMB Composite Films

Film tensile strength of films decreased 8.6% and 12.6%, respectively, after addition of two plant essential oils (Table 3), while tensile strength of increased from 15.5% to 21.6% when nano-materials were added. Simultaneous addition of nano-Ag and NBC was associated with a 41.6% increase in tensile strength, indicating that addition of NBC and nano-Ag can improve mechanical properties. There was no significant negative correlation between the tensile strength and elongation at break (*p* value is 0.51 and the correlation coefficient is −0.30, respectively), suggesting that nano-particles had different effects on the mechanical properties of the films.

It is well known that while the elongation at break of films was reduced as the increase of tensile strength of films, we see the same in this study. Compared with nano-silver, the addition of fennel essential oil and rose essential oil did not significantly change the tensile strength of the film, probably because the addition of nano-silver increased the crystallinity of the composite film, thus improving the tensile strength of the composite film. One other mechanisms beside crystallinity is that that better dispersion of rigid nano-particles could also act as reinforcing agents and possibly enhance mechanical strength of the film matrices [30].

There was no correlation between opacity and tensile strength or elongation at break (*p* value > 0.05). There is a negative correlation between the tensile strength of the composite film and the kinematic viscosity of the composite film solution (*p* value was 0.018 and correlation coefficient is −0.84, respectively). However, the kinematic viscosity of the composite film solution is mainly related to the nature of the solute and the amount of addition. Water vapor transmission rate varied with additives, but the relationship was poorly correlated. However, the kinematic viscosity of the composite film solution is mainly related to the nature of the solute and the amount of addition. However, the water vapor permeability of the films decreased by 41% after the addition of BNC to the films, probably due to the shrinkage of the film microstructure to form a dense matrix, thus reducing the diffusion of water vapor and gas molecules, resulting in a decrease in the permeability of the films [31].

Water vapor transmission rate was reduced about 42.2% with the addition of NBC. We suspect that the fine film architecture of BC and NBC differed from those of the plant-derived cellulose and these differences altered internal porosity and water diffusion. The results suggest the need for further assessments of the effects of additives on porosity.

## 4. Conclusions

Compared with cellulose from plant, BC has a larger surface area. CMBC was successfully prepared from BC. CMBC prepared from bacterial cellulose had a higher degree of substitution (*DS*) than CMC. When nano-silver and nano cellulose were added into the CMBC composite films at the same time, we measured that antibacterial activity, thermal stability, and mechanical properties were significantly improved. We hypothesize that this general improvement could result from at least a modification of the microstructure, with altered porosity and tights polymers interactions. The composite films could be further tested as a medical packaging or food packaging material, such as packaging for disposable medical supplies. In addition, it was found that the kinematic viscosity of the composite film solution and the water vapor transmission rate of the composite film decreased after the addition of BNC, probably due to the dispersing effect of BNC. Next, we will further study the antibacterial properties of CMC prepared by compounding plant cellulose and bacterial cellulose.

## Figures and Tables

**Table 1:** Degree of substitution of CMBC and CMC *.

Sample	CMBC	C-CMC	H-CMC	B-CMC
Degree of Substitution	0.81	0.64	0.64	0.65
	(0.02)	(0.01)	(0.09)	(0.05)

*: Numbers in parentheses are standard deviations.

**Table 2:** TGA thermal values and inhibition diameter of composite film made from bacterial- or plant-derived celluloses *.

Cellulose	Additive	*E. coli* (mm)	*S. aureus* (mm)	Residual Material (%)	Peak Temperature (°C)	
BC	Control	0	0	40.3	250	-
	Rose essential oil	6.26 (0.02)	6.36 (0.02)	40.3	250	-
	Fennel essential oil	6.04 (0.02)	6.13 (0.01)	36.8	250	-
	Nano-SiO_2_	0	0	42.0	250	-
	Nano-TiO_2_	7.42 (0.01)	7.75 (0.04)	45.5	250	-
	Nano-Ag	9.04 (0.11)	9.41 (0.06)	43.8	250	-
Cellulose-C	Nano-Ag	7.33 (0.32)	7.56 (0.15)	33.3	210	300
Cellulose-H	Nano-Ag	7.71 (0.16)	7.81 (0.04)	30.4	210	300
Cellulose-B	Nano-Ag	6.76 (0.04)	6.70 (0.06)	30.4	210	300
BC	Nano-Ag/NBC	13.1 (0.50)	15.0 (0.50)	45.5	250	-

*: Numbers in parentheses are standard deviations.

**Table 3:** Mechanical and physical properties of CMBC composite films *.

Additive	Tensile Strength (MPa)	Elongation at Break (%)	Opacity (A/mm)	The Kinematic Viscosity (mm^2^/s)	Water Vapor Permeability (g·cm/(cm^2^·s·Pa))
Control	28.8	19.1	8.60	277.6	0.12
	(2.23)	(2.26)	(1.11)	(2.95)	(0.02)
Rose essential oil	26.4	6.16	9.60	264.5	0.16
	(2.69)	(0.91)	(0.67)	(2.31)	(0.04)
Fennel essential oil	25.2	7.36	8.71	243.6	0.09
	(3.74)	(1.30)	(1.26)	(3.01)	(0.01)
Nano-SiO_2_	34.4	8.81	17.1	237.5	0.11
	(3.21)	(2.55)	(1.65)	(3.54)	(0.01)
Nano-TiO_2_	35.1	8.53	12.3	209.6	0.12
	(3.21)	(0.94)	(2.13)	(2.65)	(0.02)
Nano-Ag	33.3	9.36	9.07	215.7	0.12
	(3.04)	(1.07)	(1.29)	(3.00)	(0.03)
Nano-Ag/NBC	40.8	4.41	7.20	184.1	0.07
	(4.41)	(0.39)	(0.58)	(5.25)	(0.01)

*: Numbers in parentheses are standard deviations.

## Data Availability

The data presented in this study are available from the listed authors.
